# What is the Relationship Between Raising the Minimum Legal Sales Age of Tobacco Above 20 and Cigarette Smoking? A Systematic Review

**DOI:** 10.1093/ntr/ntae206

**Published:** 2024-09-05

**Authors:** Nathan Davies, Ilze Bogdanovica, Shaun McGill, Rachael L Murray

**Affiliations:** Nottingham Centre for Public Health and Epidemiology, School of Medicine, University of Nottingham, Nottingham City Hospital, Nottingham, NG5 1PB, UK; Nottingham Centre for Public Health and Epidemiology, School of Medicine, University of Nottingham, Nottingham City Hospital, Nottingham NG5 1PB, UK and SPECTRUM consortium, Edinburgh, UK; Healthcare Public Health, NHS England Midlands, Nottingham, UK; Nottingham Centre for Public Health and Epidemiology, School of Medicine, University of Nottingham, Nottingham City Hospital, Nottingham NG5 1PB, UK and SPECTRUM consortium, Edinburgh, UK

## Abstract

**Introduction:**

There is considerable interest in raising the age of sale of tobacco above the conventional age of 18 years. We systematically reviewed whether raising the minimum legal sales age of tobacco (MLSA) to 20 or above is associated with a reduced prevalence of smoking compared to an MLSA set at 18 or below.

**Aims and Methods:**

Following a preregistered protocol on PROSPERO (ref: CRD42022347604), six databases of peer-reviewed journals were searched from January 2015 to April 2024. Backward and forward reference searching was conducted. Included studies assessed the association between MLSAs ≥20 with cigarette smoking or cigarette sales for those aged 11–20 years. Assessments on e-cigarettes were excluded. Pairs of reviewers independently extracted study data. We used ROBINS-I to assess the risk of bias and GRADE to assess the quality of evidence. Findings were also synthesized narratively.

**Results:**

Twenty-three studies were reviewed and 34 estimates of association were extracted. All extracted studies related to Tobacco 21 laws in the United States. Moderate quality evidence was found for reduced cigarette sales, moderate quality evidence was found for reduced current smoking for 18–20-year-olds, and low-quality evidence was found for reduced current smoking for 11–17-year-olds. The positive association was stronger for those with lower education. Study bias was variable.

**Conclusions:**

There is moderate quality evidence that Tobacco 21 can reduce overall cigarette sales and current cigarette smoking amongst those aged 18–20 years. It has the potential to reduce health inequalities. Research in settings other than the United States is required.

**Implications:**

This systematic review on raising the minimum legal sale age of tobacco to 20 or above demonstrates there is moderate quality evidence that such laws reduce cigarette sales and moderate quality evidence they reduce smoking prevalence amongst those aged 18–20 years compared to a minimum legal sale age of 18 years or below. The research highlights potential benefits in reducing health inequalities, especially for individuals from lower educational backgrounds. Studies are limited to the United States, highlighting a need for more global research to assess the impact of these policies in other settings.

## Introduction

Globally, over 80% of tobacco smokers start smoking aged 15–24 years.^1^ In 2019, 155 million in this age group were regular tobacco smokers.^1^ Preventing both initiation and regular tobacco use in this age group, in which individuals are markedly susceptible to addiction,^2^ is critical to prevent future smoking harm. Minimum legal sales age (MLSA) laws, which prohibit retailers and vendors from selling tobacco products to those under a certain age, are one policy option for reducing access to tobacco products.

There is good historical evidence that raising the MLSA to 18 was associated with reduced smoking rates in the target population in England after implementation in 2007.^3–5^ It was also linked with reduced commercial tobacco purchases in Finland after implementation in 1995,^6^ although a study on raised European MLSAs did not find an overall association with smoking prevalence.^7^ Given the evidence base for raised MLSAs, and the uniquely harmful properties of tobacco, there has been renewed global interest in increasing MLSAs beyond 18.^8–12^ Article 16 of the World Health Organization Framework Convention for Tobacco Control compels signatories to prohibit the sale of tobacco products to minors but does not specify an exact age limit.^13^

The first Tobacco 21 (T21) law in the 21st century was introduced in Needham, Massachusetts. Following this, local areas, cities, and states across the United States began to introduce Tobacco 21 (T21) laws^14^ culminating in a national law being passed in 2019.^15^ Several other countries, including Ethiopia, Honduras, Japan, Kazakhstan, Mongolia, Philippines, Singapore, Sri Lanka, Thailand, Turkmenistan, and Uganda have been reported to introduce an MLSA of at least 20 in recent years.^16^ In 2024, the Labour government of the United Kingdom announced plans to adopt a law banning the sale of tobacco to anyone born after 2009.^17^ This policy, known as the smoke-free generation policy, had been tabled by the previous Conservative government but not passed before a general election.^18^ New Zealand adopted similar legislation in 2021^19^ but a new government reversed the legislation before it could be implemented.^20^ There have also been issues with the implementation of smoke-free generation laws in Malaysia, due to last-minute changes in the Control of Smoking Products for Public Health Bill,^21^ and in Denmark, where the European Union tobacco directive has been cited as a potential legal roadblock.^22^

As policymakers across the globe continue to consider raising MLSA laws as an important component of strategies to reduce tobacco harm, understanding the effectiveness of such policies is of great importance. The main objective of this systematic review was to determine if raising the legal age of sale of tobacco to 20 or above is associated with reduced prevalence of smoking amongst those aged 11–20, compared to a legal age of tobacco set at 18 or below.

## Methods

This systematic review was conducted in line with a preregistered protocol on PROSPERO (ref: CRD42022347604) and the Preferred Reporting Items for Systematic Review and Meta-Analyses (PRISMA) reporting guidelines.^23^

### Selection Criteria

Studies were eligible if they reported the effect on cigarette use of raising the MLSA to 20 or above. They were eligible if the study population included children and young people aged 11–25 years, or if data restricted to this age group could be extracted from the broader study. We excluded studies where an MLSA of 20 or above was introduced where no prior age-of-sale limit previously existed. We excluded qualitative studies and studies that purely reported estimates relating to e-cigarettes. There were no geographical restrictions.

### Search Strategy and Study Selection

We searched the electronic databases Embase through OVID, MEDLINE through PubMed, PyscINFO through Ovid, ProQUEST Public Health, ProQUESTION Dissertations and Theses, and CINAHL through Ebscohost, for studies published from January 1, 2015 (the year in which the first study evaluating the local T21 law in Needham, Massachusetts, was published) to April 18, 2024. A full list of search terms is provided in Supplementary File. No restrictions were in place for the observational period or language. Records were extracted into Rayyan^24^ and de-deduplicated. Two of the three reviewers (ND, IB, and RM) screened titles and abstracts, and subsequently full texts, to identify eligible studies. ND hand-searched reference lists of identified studies to identify any additional studies. The conflict was settled by discussion or by adjudication from the third screener.

### Data Extraction and Quality Assessment

A standard data extraction form was piloted and used to record details for each eligible study by two reviewers (ND and IB). Two of three reviewers (ND, RM, and SM) independently assessed quality using the Risk of Bias in Non-randomized Studies—of Interventions (ROBINS-I) assessment tool^25^ with conflict resolved by discussion and adjudication from a third reviewer where necessary. Studies were given a risk of bias score for seven domains between “low” and “critical” and given an overall risk of bias score.

### Data Synthesis

We planned to conduct a meta-analysis and related sensitivity analyses. However, the data was not found to be suitable for meta-analysis. Even after approaching authors for further information, many studies eligible for inclusion used measures of effect that could not be harmonized, including several studies with a moderate risk of bias. Furthermore, studies included very heterogeneous population groups, comparators, and outcome measures.

Instead, GRADE criteria^26^ were used to report confidence in overall estimates of the effect for cigarette sales and for current smoking in two preplanned subgroups, those aged 18–20 and those aged under 18 years. A narrative synthesis was also conducted. This followed the process of developing a preliminary synthesis, exploring relationships within and between studies through visual tabulation of intervention type, population characteristics, measures of effect, and whether a statistically significant association was found between current smoking or cigarette sales and T21. The robustness of the synthesis was assessed by considering the quality of included studies.^27^ As no primary patient-level data was used, ethical approval was not required.

## Results

### Overview of Included Studies

**Figure 1.**
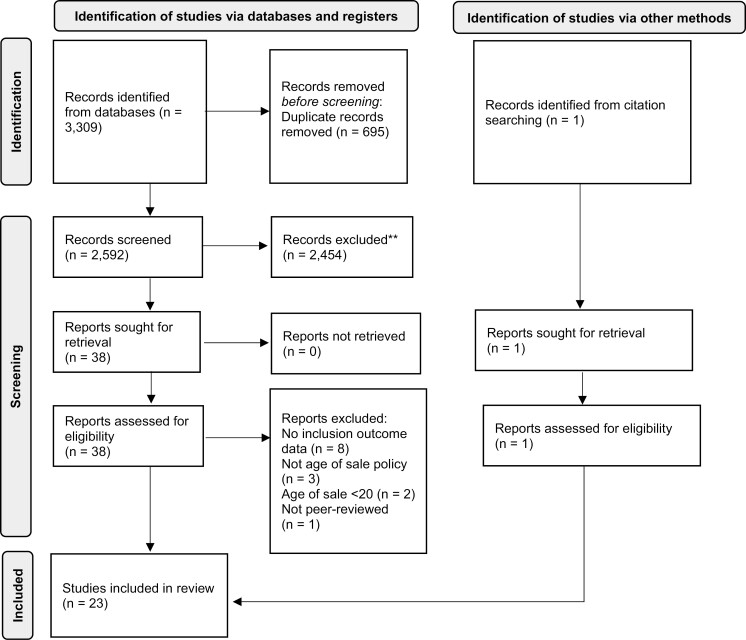
PRISMA flow diagram.

Database searches identified 3309 papers, of which 2592 were unique papers. Thirty-eight papers remained after title and abstract screening. Fifteen were judged ineligible in the full-text review; eight papers had no outcome data, four papers did not relate to age-of-sale policy, two papers related to age-of-sale policies restricting sales to those under 20 only, and one was not peer-reviewed. In total, 23 papers were included (Figure 1).

Thirty-four estimates of the association of the policy with current smoking or cigarette sales were extracted from the 23 included studies^28–50^ (Tables 1 and 2). Full data extraction is available at https://www.doi.org/10.17605/OSF.IO/8D4TB.

**Table 1.** Included Estimates of Association by Design, Intervention Level, Age Group, Population Size, and Risk of Bias

Study author	Design	Intervention level	Intervention age group	Sample size	Risk of bias
Friedman 2019^28^	One-off cross-sectional	Local and state	18–20 year olds	1869	Serious
Macinko 2018^29^	Repeated cross-sectional	City	7–12 graders	76 668	Moderate
Macinko 2018^29^	Repeated cross-sectional	City	9–12 graders	71 214	Serious
Garcia-Ramirez 2022^30^	Repeated cross-sectional	State	7, 9, and 11 graders	2 229 401	Moderate
Friedman 2020^31^	Repeated cross-sectional	Local	18–20 year olds	25 066	Moderate
Agaku 2022^32^	One-off cross-sectional	State	18–20 year olds	10 146	Serious
Agaku 2022^32^	One-off cross-sectional	State	9–12 graders	182 491	Serious
Dove 2021^33^	Repeated cross-sectional	State	18-20 year olds	15 863	Serious
Colston 2022^34^	Repeated cross-sectional	Local, county, and state laws	8 graders	92 922	Moderate
Colston 2022^34^	Repeated cross-sectional	Local, county, and state laws	10 graders	88 628	Moderate
Colston 2022^34^	Repeated cross-sectional	Local, county, and state laws	12 graders	81 082	Moderate
Roberts 2022^35^	Cohort study	City	First-year university	1140	Critical
Grube 2021^36^	Repeated cross-sectional	State	7, 9, and 11 graders	2 956 054	Moderate
Glover-Kudon 2021^37^	Longitudinal cigarette sales data	State (Hawaii)	N/A	N/A	Serious
Glover-Kudon 2021^37^	Longitudinal cigarette sales data	State (California)	N/A	N/A	Serious
Schneider 2016^38^	Repeated cross-sectional	Local	9–12 graders	16 385 to 17 089	Critical
Schiff 2021^39^	Cohort study	State	19–20 year olds	3111	Critical
Hawkins 2022^40^	Repeated cross-sectional	County	9–12 graders	9988	Moderate
Liber 2022^41^	Longitudinal cigarette sales data	Designated market areas	N/A	N/A	Moderate
Ali 2020^42^	Longitudinal cigarette sales data	State (California)	N/A	N/A	Serious
Ali 2020^42^	Longitudinal cigarette sales data	State (Hawaii)	N/A	N/A	Serious
Patel 2023^43^	Cohort study	Local, city, or state	15–21 year olds	13 990	Moderate
Wilhelm 2021^44^	Repeated cross-sectional	City or county	8 and 9 graders	210 177	Serious
Wilhelm 2021^44^	Repeated cross-sectional	City or county	11 graders	210 177	Serious
Yan 2014^45^	Repeated cross-sectional	State	20 year olds	60 710	Moderate
Trapl 2022^46^	Repeated cross-sectional	City	9–12 graders	12 616	Moderate
Abouk 2024^49^	Repeated cross-sectional	53 918	8 and 10 graders	53 918	Moderate
Abouk 2024^49^	Repeated cross-sectional	21 516	12 graders	21 516	Moderate
Abouk 2024^49^	Longitudinal cigarette sales data	N/A	N/A	N/A	Serious
Hansen 2023^50^	Repeated cross-sectional	95 557	18–20 year olds	95 557	Moderate
Hansen 2023^50^	Repeated cross-sectional	87 612	18–20 year olds	87 612	Moderate
Tennekoon 2023^47^	Repeated cross-sectional	State	18–20 year old (pregnant)	2 140 600	Serious
Friedman 2024^48^	Cohort study	Local, substate, state	18–20 year olds	55 775	Serious
Friedman 2024^48^	Repeated cross-sectional	Local, substate, state, federal	18–20 year olds	Unknown	Serious

^*^per survey, four surveys.

**Table 2.** Included Estimates of Association by Age Group, Sample Size, Outcome, Direction of Association, and Risk of Bias

Study author	Intervention age group	Sample size	Outcome	Direction of association	Risk of bias
Friedman 2019^28^	18–20 year olds	1869	Current cigarette smoking: AOR 0.6 (0.39, 0.92)^	Favors intervention	Serious
Macinko 2018^29^	7–12 graders	76 668	Current cigarette smoking: APR 1.25 (0.88, 1.76)	Not significant	Moderate
Macinko 2018^29^	9–12 graders	71 214	Current cigarette smoking: APR 1.40 (1.10, 1.80)^	Favors control	Serious
Garcia-Ramirez 2022^30^	7, 9, and 11 graders	2 229 401	Current cigarette smoking: AOR 0.98 (0.94, 1.03)	Not significant	Moderate
Friedman 2020^31^	18-20 year olds	25,066	Current cigarette smoking: −0.0306 absolute risk reduction (CI −0.0548 to −0.0063)^	Favors intervention	Moderate
Agaku 2022^32^	18–20 year olds	10 146	Current cigarette smoking: APR 0.58 (0.39, 0.74)^	Favors intervention	Serious
Agaku 2022^32^	9–12 graders	182 491	Current cigarette smoking: APR 0.70 (0.52, −0.93)^	Favors intervention	Serious
Dove 2021^33^	18–20 year olds	15 863	Current cigarette smoking: AOR 1.01 (0.76, 1.34)	Not significant	Serious
Colston 2022^34^	8 graders	92 922	Current cigarette smoking: ARR 0.91 (0.69, 1.20)	Not significant	Moderate
Colston 2022^34^	10 graders	88 628	Current cigarette smoking: ARR 0.96 (0.75, 1.23)	Not significant	Moderate
Colston 2022^34^	12 graders	81 082	Current cigarette smoking: ARR 0.74 (0.60 to 0.91)^	Favors intervention	Moderate
Roberts 2022^35^	First-year university students	1140	Current cigarette smoking: 4.1% (interv) 6.6% (control)	Not significant	Critical
Grube 2021^36^	7, 9, and 11 graders	2 956 054	Current cigarette smoking: AOR 0.99 (0.97, 1.01)	Not significant	Moderate
Glover-Kudon 2021 (Hawaii)^37^	N/A	N/A	Change in average monthly unit sales: intervention −4.4%, control −10.6%	Not tested	Serious
Glover-Kudon 2021 (California)^37^	N/A	N/A	Change in average monthly unit sales: intervention −11.7%, control −10.6%	NA	Serious
Schneider 2016^38^	9–12 graders	16 385 to 17,089*	Current cigarette smoking: 12.9% to 6.7% (intervention) 14.8% to 12.0% (control); *p* < .01^	Favors intervention	Critical
Schiff 2021^39^	19–20 year olds	3111	Past 30-day use, cigarette: Pre T21 = 150 (9.6%) Post T-21 = 164 (11.1%)	Not tested	Critical
Hawkins 2022^40^	9–12 graders	9988	Current cigarette smoking: Inflation model 0.12 (−1.34 to 0.11)	Not significant	Moderate
Liber 2022^41^	N/A	N/A	Absolute adjusted risk difference: Cigarette brand sales: −0.000156 (*p* = < .001)^	Favors intervention	Moderate
Ali 2020 (California)^42^	N/A	N/A	Absolute adjusted risk difference of cigarette sales: −9.41 (−15.52, −3.30)^	Favors intervention	Serious
Ali 2020 (Hawaii)^42^	N/A	N/A	Absolute adjusted risk difference of cigarette sales: −0.57 (−0.83, −0.30)^	Favors intervention	Serious
Patel 2023^43^	15–21 year olds	13 990	Current cigarette smoking: AOR 0.90 (CI: 0.72 to 1.14)	Not significant	Moderate
Wilhelm 2021^44^	8 and 9 graders	210 177	Current cigarette use, 8/9 grade: AOR 0.81 (0.67, 0.99)^	Favors intervention	Serious
Wilhelm 2021^44^	11 graders	210 177	Cigarettes, 11 grade: AOR 1.2 (0.97, 1.48)	Not significant	Serious
Yan 2014^45^	20 year olds	60 710	Prenatal smoking: 0.013 (SE = 0.010)	Not significant	Moderate
Trapl 2022^46^	9–12 graders	12 616	Adjusted current cigarette smoking: (β = 0.04 [SE = 0.07]; *p* = .56)	Not significant	Moderate
Abouk 2024^49^	8 and 10 graders	53 918	Cigarette use past month: −.0100 (SE = 0.0071) (Table 3, column 2)	Not significant	Moderate
Abouk 2024^49^	12 graders	21 516	Cigarette use past month: −0.0208 (SE = 0.0100), *p* = < .05	Favors intervention	Moderate
Abouk 2024^49^	N/A	N/A	Cigarette sales: −.00714 (0.0334) (*p* < .05)	Favors intervention	Serious
Hansen 2023^50^	18–20 year olds	95 557	Smoking participation: −0.037 (SE = 0.009), *p* = < .01	Favors intervention	Moderate
Hansen 2023^50^	18–20 year olds	87 612	Smoking participation: −0.009 (SE = 0.019)	Not significant	Moderate
Tennekoon 2023^47^	18–20 year old (pregnant)	2 140 600	Current smoking at the beginning of pregnancy: −0.0574 (0.0009), *p* ≤ .01	Favors intervention	Serious
Friedman 2024^48^	18–20 year olds	55 775	Current cigarette use 0.60 (CI 0.45, 0.79), *p* < .01	Favors intervention	Serious
Friedman 2024^48^	18–20 year olds	Unknown	Current established smoking: 0.38 (0.25, 0.57)	Favors intervention	Serious

(AOR = adjusted odds ratio, APR = adjusted prevalence ratio, ARR = adjusted risk ratio) * per survey, four surveys ^ = statistically significant at *p* ≤ .05.

The detailed results of the ROBINS-I assessments are set out in Supplementary File. These assessments relate specifically to single effect estimates, not entire studies. Estimates are very unlikely to be graded at a lower risk of bias than “moderate” using the ROBINS-I tool, given residual confounding is almost inevitably introduced in non-randomized studies.^25^ Sixteen estimates were judged to be of moderate overall risk of bias, 15 of serious risk of bias, and three of critical risk of bias. Sixteen estimates found a statistically significant association favoring T21, 14 estimates were not significant, one estimate favored the control (MLSA of tobacco remaining at 18) and three estimates did not assess statistical significance.

Twenty-eight estimates were based on self-reported current smoking, of which 21 were based on repeated cross-sectional studies, three on one-off cross-sectional studies, and four were based on cohort studies. Six of the estimates were based on cigarette sales data over time.

Using GRADE criteria following the use of ROBINS-I^26^ we found moderate quality evidence that Tobacco 21 laws reduce overall cigarette sales and that they cause a reduction in current smoking specifically amongst 18–20-year-olds. We found low-quality evidence that Tobacco 21 laws reduce smoking rates amongst 11–17-year-olds (Table 3). All three estimates of quality were downgraded from “high” by the risk of study bias, and evidence for 11–17-year-old current smoking rates was also downgraded by the inconsistency of results.

**Table 3.** GRADE Assessment for Tobacco 21

	Estimates of effect	Quality of evidence	Factors that reduce the quality of evidence	Factors that increase the quality of evidence	Published studies [reference #]
Reduction in current smoking amongst 18–20-year-olds	12	Moderate	• Risk of bias	None	Friedman (2019); Friedman (2020); Agaku (2022); Dove (2021); Roberts (2022); Schiff (2021); Yan (2014); Abouk (2024); Hansen (2023); Tennekoon (2023); Friedman 2024 ^[28,31–33,35,39,45,47–50]^
Reduction in current smoking amongst 11–17 year olds	15	Low	• Risk of bias • Inconsistency of results	None	Macinko (2018); Garcia-Ramirez (2022); Agaku (2022); Colston (2022); Grube (2022); Schneider (2016); Hawkins (2022); Wilhelm (2021); Trapl (2022); Abouk (2022) ^[29,30,32,34,36,38,40,44,46,49]^
Reduction in cigarette sales	6	Moderate	• Risk of bias	None	Glover-Kudon (2021); Liber (2021); Ali (2020); Abouk (2024) ^[37,41,42,49]^

GRADE Working Group grades of evidence:.

*High quality:* Further research is very unlikely to change our confidence in the estimate of effect.

*Moderate quality:* Further research is likely to have an important impact on our confidence in the estimate of effect and may change the estimate.

*Low quality:* Further research is very likely to have an important impact on our confidence in the estimate of effect and is likely to change the estimate.

*Very low quality:* We are very uncertain about the estimate.

### Single Law and Multiple Law Evaluation

Narratively, 18% of analyses evaluating the impact of a single local, city, or state law reported a positive association between T21 and reduced current smoking rates, with 11 of the 17 analyses at serious or critical risk of bias.^29,30,33,35–40,42,44–46^ This includes the study of New York’s T21 law, which conducted the only analysis to find an association between increased smoking rates and T21.^29^ However, analyses that evaluated T21 across multiple T21 laws in multiple jurisdictions found significant associations between reduced current cigarette smoking and T21 on 12 of the 17 occasions (71%), with all analyses at moderate or serious risk of bias.^28,31,32,34,41,43,47–50^

### Consideration of Health Inequalities

Six analyses specifically discussed associations of T21 by ethnicity or race in results. The findings were extremely heterogeneous. Agaku^32^ and Yan^45^ found that White non-Hispanics were most likely to benefit from T21 laws, Colston^34^ found Hispanic non-White and other/mixed groups were more likely to benefit, Hansen^50^ that Black groups were most likely to benefit, Abouk^49^ that Other groups were more likely to benefit, and Trapl^46^ found inequity between Black/Hispanic groups and White groups was reduced.

Two analyses at moderate risk of bias considered the differential associations of T21 by education.^34,45^ Both found that T21 was associated more strongly with reduced cigarette smoking for those with lesser parental or personal education than those with greater parental or personal education.

One study at moderate risk of bias (Garcia-Ramirez) considered differential associations of T21 on current smoking by sexual minority status and found little difference between groups.^30^

## Discussion

To the best of our knowledge, this is the first systematic review assessing the impact of laws raising the MLSA to 20 or above on smoking rates. Studies displayed a high level of heterogeneity in analytical approaches, population groups, and the types of laws enforced.

Our systematic review identified evidence of moderate quality that supports the introduction of T21 laws to reduce cigarette sales. When considering studies with defined age groups, evidence of the policy having an impact on young adults directly covered by the law (18–20-year-olds) was of moderate quality, whilst the evidence that it impacts children indirectly affected (11–17-year-olds) was of low quality. This is supplemented by two recent studies, not included in this systematic review because they did not include data on smoking rates or cigarette sales. One found lower smoking *intentions* amongst those with knowledge of T21 laws^51^ and a separate study found that the proportion of youth who perceived easy access to cigarettes significantly decreased following the federal T21 law.^52^

All studies included in this review were conducted in the United States. One study on retail compliance with the T20 law implemented in Thailand in 2017 which was excluded found that 38% of retailers self-reported selling cigarettes to those under 20s post-implementation.^53^

There were no studies at low risk of bias, which was expected given that the ROBINS-I assessment tool is extremely unlikely to assess non-randomized studies to be at low risk of bias. There were, however, 34 separate analyses included in this review, which is a relatively rich source of evidence for a single tobacco control policy.

Age appears to mediate the association with T21. The impact of T21 on the ability of 18–20-year-olds and older teens to purchase tobacco or obtain it from peers may be more immediate.^51^ For younger groups, it is possible that more time is required for T21 policies to change smoking habits, as the policy mechanism may be more reliant on disrupting the supply of tobacco from older groups and wider changes in social norms.^37^

We found that studies which included multiple T21 laws were more likely to find an association than those that evaluated a single law. Many of the early states and areas that introduced T21 and subsequently evaluated in isolation were already leaders in tobacco control, with relatively low prevalence rates amongst young people, and thus reducing cigarette smoking further is challenging. Many of the studies that focused on multiple areas included the impact of laws in parts of the country which had higher smoking prevalence and thus the scope for reduction in current smoking may have been greater.

There were encouraging findings relating to T21’s potential for reducing educational inequality in smoking, important given the extreme inequity of the impact of tobacco on health.^1,54,55^ In two analyses at moderate risk of bias, the policy was shown to have reduced disparities in smoking rates between educational groups, despite evidence there is a lower likelihood of ID checks in poorer areas.^56^ Studies reported heterogeneous differences across racial and ethnic groups, but crucially there was no evidence that white non-Hispanic groups were more likely to benefit from the policy than any other group.

It is important to note that in the United States, rapid and significant increases in e-cigarette use took place during the duration of many of the included studies, along with falls in smoking rates. Only 1.5% of those in grades 9–12 were current cigarette users in 2021.^57^ Systematically reviewing the literature on the association of T21 laws on e-cigarette use is beyond the scope of this review, but mixed results have been reported. It is possible that this rise affected the impact of T21 laws, although some analyses controlled for e-cigarette use.^30,44^ The relationship between MLSAs and e-cigarette use needs careful consideration given global increases in e-cigarette use in younger age groups.

T21 is not the only option for countries considering policies based on age of sale. The smoke-free or tobacco-free generation approach, in which the MLSA is effectively raised by one year every year, is a prominent alternative. The United Kingdom has started the process of implementing smoke-free generation laws^18^ and there are published modeling studies to support smoke-free generation laws in multiple settings in Oceania.^58–60^

### Limitations

We were not able to conduct a meta-analysis which would have provided quantified measures of association and uncertainty. However, the reporting of GRADE assessments and narrative synthesis support an understanding of the strength of evidence and populations for which T21 is likely to have the greatest effect on smoking rates.

It was not clear from most studies how roll-your-own tobacco was categorized, although survey methodologies suggested this would have largely been included under cigarette smoking. This review does not consider e-cigarettes, cigars, or smokeless tobacco products. This may have prevented useful further context on product type; for example, Trapl et al.’s paper found a significant reduction in cigar use in Cleveland compared to surrounding areas, though not cigarettes.^46^ However, given a proliferation of estimates across product types, a focus on current cigarette smoking enabled a systematic, transparent approach to synthesizing data for the outcome of greatest global relevance, given cigarette smoking is the most common form of tobacco use in this age group.^1^

Despite having no restriction on geographical location, all included studies were conducted in the United States, and nearly all focused on subnational laws or included a very short window following federal implementation. Research into Tobacco 21 at the federal level in the United States and of MLSAs above 20 in several other countries which have implemented them will be critical to inform wider global tobacco control policymaking.

We did not look in-depth at the design and enforcement of laws and how this affected estimates, which is particularly important for MLSAs.^61^ For example, California’s law exempted those serving in the military,^62^ some studies found that T21 implementation was accompanied by a lack of retailer monitoring and enforcement,^29,63^ and one study found that possession, use, or purchase laws criminalizing young people dampened the effects of T21.^48^

## Conclusions

This review demonstrates that there is moderate quality evidence that T21 policies are associated with both reducing cigarette sales overall and cigarette sales specifically in those aged 18–20. T21 may be more likely to achieve its policy goals when implemented in areas with higher current cigarette smoking rates. T21 may have a greater impact on groups with lower educational status, signifying a possible role in reducing health inequalities. Countries and regions with lower MLSAs should consider raising the age of sale of tobacco as part of a broader tobacco control strategy, paying careful attention to the design, implementation, and enforcement of new laws.

## Supplementary material

Supplementary material is available at *Nicotine and Tobacco Research* online.

ntae206_suppl_Supplementary_Material

## Data Availability

The data underlying this manuscript is available at https://www.doi.org/10.17605/OSF.IO/8D4TB
