# Correction to: What is the Relationship Between Raising the Minimum Legal Sales Age of Tobacco Above 20 and Cigarette Smoking? A Systematic Review

**DOI:** 10.1093/ntr/ntae239

**Published:** 2024-12-07

This is a correction to:

Nathan Davies, Ilze Bogdanovica, Shaun McGill, Rachael L Murray, What is the Relationship Between Raising the Minimum Legal Sales Age of Tobacco Above 20 and Cigarette Smoking? A Systematic Review, *Nicotine & Tobacco Research*, 2024;, ntae206, https://doi.org/10.1093/ntr/ntae206

In the originally published version of this manuscript, Reference 17 was not included. Instead, the reference that should have been reference 18 was included as reference 17.

Reference 17 should read:

Independent, UK. Labour revives plans to phase out smoking with Tobacco and Vapes Bill. https://www.independent.co.uk/news/uk/chris-whitty-smoking-bill-government-nhs-b2581229.html. Accessed 30 July 2024.

This error has been corrected and all subsequent references have shifted over, correctly.

Additionally, page 3 and 6 highlight minor referencing issues – on Page 3, in the “Consideration of Health Inequalities” section, and on Page 6, in the ”Discussion” section, “Three analyses” should be “Two analyses”. This error has been corrected.

