# Supplementary material for: What is the Relationship Between Raising the Minimum Legal Sales Age of Tobacco Above 20 and Cigarette Smoking? A Systematic Review
Source: Nicotine Tob Res. 2024 Sep 5;27(3):369–77. doi: 10.1093/ntr/ntae206 (PMC11847775; doi:10.1093/ntr/ntae206)
Supplement: ntae206_suppl_Supplementary_Material [file ntae206_suppl_supplementary_material.docx]

**Supplementary File**

**Tobacco 20+ systematic review search strategy**

**Search strategy for MEDLINE through PubMed**

**(**Search: **((1. Tobacco OR exp. smoking OR cigarette* OR cigar* OR smok* OR nicotine*) AND (2. "age of sale" OR "age-of-sale" OR "sale age" OR "MLA" OR "MLSA" OR "minimum legal age" OR "minimum age" OR "age of legal access" OR "sale age" OR "age of purchase" OR "purchase age" OR "minimum purchas*" OR "legal minimum age" OR "age policy" OR "T21" OR "Tobacco 21" OR "MLA" OR "MLSA" OR "legal age" OR "age restriction")) AND (("2015/01/01"[Date - Publication] : "3000"[Date - Publication]))**

**Search strategy for EMBASE through OVID**

1. **(**Tobacco or smoking or cigarette* or cigar* or smok* or nicotin*).mp. [mp=ti, ab, hw, tn, ot, dm, mf, dv, kf, fx, dq, tc, id, tm]
2. age of sale or age-of-sale or sale age or MLA or MLSA or minimum legal age or minimum age or age of legal access or sale age or age of purchase or purchase age or minimum purchas* or legal minimum age or age policy or T21 or Tobacco 21 or MLA or MLSA or legal age or age restriction
3. limit 2 to yr=”2015-Current”
4. 1 and 2 and 3

**Search strategy for PyschInfo (through OVID)**

1. (Tobacco or smoking or cigarette* or cigar* or smok* or nicotin*).mp. [mp=ti, ab, hw, tn, ot, dm, mf, dv, kf, fx, dq, tc, id, tm]
2. age of sale or age-of-sale or sale age or MLA or MLSA or minimum legal age or minimum age or age of legal access or sale age or age of purchase or purchase age or minimum purchas* or legal minimum age or age policy or T21 or Tobacco 21 or MLA or MLSA or legal age or age restriction
3. limit 2 to yr=”2015-Current”
4. 1 and 2 and 3

**Search strategy for ProQuest Public Health Database and Dissertations and Theses**

(Tobacco OR exp. smoking OR cigarette* OR cigar* OR smok* OR nicotin*) AND ("age of sale" OR "age-of-sale" OR "sale age" OR "MLA" OR "MLSA" OR "minimum legal age" OR "minimum age" OR "age of legal access" OR "sale age" OR "age of purchase" OR "purchase age" OR "minimum purchas*" OR "legal minimum age" OR "age policy" OR "T21" OR "Tobacco 21" OR "MLA" OR "MLSA" OR "legal age" OR "age restriction") Date: after 01 January 2015

**Search strategy for CINHL through Ebscohost**

| ( 1. Tobacco OR exp. smoking OR cigarette* OR cigar* OR smok* OR nicotin* ) AND ( 2. “age of sale” OR “age-of-sale” OR “sale age” OR “MLA” OR “MLSA” OR “minimum legal age” OR “minimum age” OR “age of legal access” OR “sale age” OR “age of purchase” OR “purchase age” OR “minimum purchas*” OR “legal minimum age” OR “age policy” OR “T21” OR “Tobacco 21” OR “MLA” OR “MLSA” OR “legal age” OR “age restriction ) |  |
| --- | --- |

Limiters - Published Date: 20150101-20241231

Note: ScienceDirect was not searched as originally planned in PROSPERO.

**Full risk of bias assessment**

| **Paper** | **Intervention location** | **Numerical result being assessed** | **Confounding** | **Participant selection** | **Classification of interventions** | **Deviation from intended intervention** | **Missing data** | **Measurement of outcomes** | **Selection of reported result** | **Overall risk of bias** |
| --- | --- | --- | --- | --- | --- | --- | --- | --- | --- | --- |
| **Abouk 2024** | Various localities | 8/10 grader: -.0100 (SE = 0.0071) (Table 3, column 2) | Moderate | Moderate | Low | Low | Moderate | Low | Low | **Moderate** |
| **Abouk 2024** | Various localities | 12 grader, -0.0208 (SE = 0.0100), p = <0.05 (Table 4, column 2) | Moderate | Moderate | Low | Low | Moderate | Low | Low | **Moderate** |
| **Abouk 2024 sales** | Various localities | -.00714 (0.0334), p<0.05 (Table 10, column 1, row 1) | Serious | Low | Low | Low | Low | Low | Low | **Serious** |
| **Ali 2022** | Hawaii | Versus intervention states: -0.57 (-0.83 to -0.30) (Table 2) | Serious | Low | Low | Low | Low | Low | Low | **Serious** |
| **Ali 2022** | California | Versus intervention states: -9.41 (-15.52 to -3.30) (Table 2) | Serious | Low | Low | Low | Low | Low | Low | **Serious** |
| **Agaku 2022 YRBS** | States implementing T21 | OR 0.70 (CI 0.52-0.93) (Table 4) | Serious | Low | Moderate | Low | NI | Low | Low | **Serious** |
| **Agaku 2022 BRFSS** | States implementing T21 | OR 0.58 (0.39 - 0.74) (Table 3) | Serious | Serious | Moderate | Low | NI | Low | Low | **Serious** |
| **Colston 2022** | Covered by local, county or state law | Smoking participation for 8 grader: ARR 0.91 (0.69, 1.20) (with multiple imputation) (Table 2) | Moderate | Low | Low | Low | Low | Low | Moderate | **Moderate** |
| **Colston 2022** | Covered by local, county or state law | Smoking participation for 10 grader = ARR 0.91 (0.69, 1.20) (with multiple imputation) (Table 2) | Moderate | Low | Low | Low | Low | Low | Moderate | **Moderate** |
| **Colston 2022** | Covered by local, county or state law | Smoking participation for 12 grader = ARR 0.74 (0.60,0.91) (with multiple imputation) (Table 2) | Moderate | Low | Low | Low | Low | Low | Moderate | **Moderate** |
| **Friedman 2019** | Various localities | OR 0.61 (95% CI 0.42, 0.89) Table 3, column 2, row 3 | Serious | Serious | Low | Low | Low | Low | Low | **Serious** |
| **Friedman 2020** | Various localities | Tobacco-21 policy covering entire MMS = -0.0306 reduction in 18-20 year olds (CI -0.0548 to -0.0063) - difference in difference, 18-20 (para 3 of results, top-left cell Table 2) | Moderate | Low | Moderate | Low | Low | Low | Low | **Moderate** |
| **Friedman 2024 PATH** | Various localities | Current cigarette use 0.60 (CI 0.45, 0.79), p < 0.01 (Figure 1) | Serious | Low | Low | Low | NI | Low | Low | **Serious** |
| **Friedman 2024 BRFSS** | Various localities | AOR 0.38 (0.25 - 0.57) (Table 1, column 4) | Serious | Low | Low | Low | NI | Low | Low | **Serious** |
| **Garcia-Ramirez 2022** | California | OR 0.98 (0.94, 1.03) (Table 1, Model 1, T21) | Moderate | Low | Low | Low | Moderate | Low | Low | **Moderate** |
| **Glover-Kudon 2021** | California | California −11.7%**, USA −10.6%** (Table 1) | Moderate | Serious | Low | Low | Low | Low | Low | **Serious** |
| **Glover-Kudon 2021** | Hawaii | Hawaii −4.4%**, USA −10.6%** (Table 1) | Moderate | Serious | Low | Low | Low | Low | Low | **Serious** |
| **Grube 2021** | California | Past 30-day cigarette smoking: OR 0.99 (0.97 - 1.01) (Table 1, Column 3) | Moderate | Low | Low | Low | Moderate | Low | Low | **Moderate** |
| **Hansen 2023 YRBS** | Various localities | -0.009 (SE = 0.019) (Table 5, column 6) | Moderate | Low/low | Low | Low | Moderate | Low | Low | **Moderate** |
| **Hansen 2023 BRFSS** | Various localities | -0.037 (SE = 0.009), p = <0.01 (Table 1, column 6) | Moderate | Low | Low | Low | Moderate | Low | Low | **Moderate** |
| **Hawkins 2022** | Localities in Massachusetts | 0.12 (CI = -1.34 to 0.11) (table 1, row 2, inflation model) | Moderate | Low | Low | Low | Moderate | Low | Low | **Moderate** |
| **Liber 2022** | Various | Diff-in-diff change in disproportionately young brands : −0.00156 (p = <0.001) (Table 3) | Moderate | Moderate | Low | Low | Low | Low | Low | **Moderate** |
| **Macinko 2018** | New York (vs New York state) | APR 1.25 (0.88, 1.76) (Table 3, column 2) | Moderate | Low | Serious | Low | Moderate | Low | Low | **Serious** |
| **Macinko 2018** | New York (vs FL counties) | APR 1.40 (1.10, 1.80) (Table 3, column 2) | Moderate | Low | Low | Low | Moderate | Low | Low | **Moderate** |
| **Roberts 2022** | Columbus, Ohio | 1st years 2016 (comparator) - 6.6%. 1st year 2018 (intervention) - 4.1%. (Table 2) | Critical | Serious | Low | Low | Moderate | Low | Low | **Critical** |
| **Patel 2022** | Various localities | 0.90 (CI 0.72,1.14) Table 3, Model 1 | Moderate | Moderate | Low | Low | Moderate | Low | Low | **Moderate** |
| **Schneider 2015** | Needham, Massachusetts | -1.08, p<0.001 (Table 1, column 1) | Serious | Serious | NI | Low | Moderate | Low | Serious | **Serious** |
| **Schiff 2021** | California | Pre T21 = 150 (9.6%) Post T-21 = 164 (11.1%) (Table 1) | Critical | Low | Low | Low | Serious | Low | Low | **Critical** |
| **Tennekoon 2023** | Various localities | -0.0574 (0.0009), p = <0.01 | Serious | Low | Moderate | Low | NI | Low | Low | **Serious** |
| **Trapl 202** | City of Cleveland | (β = 0.04 [SE, 0.07]; P=.56) (Table 3, row 1) | Moderate | Low | Moderate | Low | Moderate | Low | Low | **Moderate** |
| **Wilhelm 2022** | Minnesota T21 area | Current cigarette use, 8/9 grade: AOR 0.81 (0.67, 0.99) (Table 2) | Serious | Moderate | Low | Low | Serious | Low | Moderate | **Serious** |
| **Wilhelm 2022** | Minnesota T21 area | Cigarette use, 11 grade: 1.2 (0.97, 1.48) (Table 2) | Serious | Moderate | Low | Low | Serious | Low | Moderate | **Serious** |
| **Yan 2014** | Pennsylvania | Prenatal smoking: 0.013 (0.010) (Table 2, column 8) | Moderate | Low | Low | Moderate | Low | Moderate | Low | **Moderate** |
